# Symptomatic sinus bradycardia during the recovery phase of high-altitude pulmonary edema: a case report

**DOI:** 10.3389/fmed.2026.1754071

**Published:** 2026-01-30

**Authors:** Jiale Xie, Weiping Li, Jia Luan, Zhibiao Cai, Haijun Miao

**Affiliations:** 1 The 940th Hospital of Joint Logistic Support Force of Chinese PLA, Lanzhou, Gansu, China; 2 The 944th Hospital of Joint Logistic Support Force of Chinese PLA, Jiuquan, Gansu, China

**Keywords:** autonomic dysfunction, bradycardia, glucocorticoids, high-altitude pulmonary edema, hypokalemia

## Abstract

This case report describes a 28-year-old healthy male who developed high-altitude pulmonary edema (HAPE) after a rapid ascent. While his condition improved with standard therapy (including corticosteroids, diuretics, and supplemental oxygen), he developed symptomatic sinus bradycardia (heart rate nadir: 37 bpm) 2 days after discontinuation of these medications. The bradycardia resolved with intravenous potassium supplementation and fluid resuscitation, and his heart rate recovered to 59 bpm. This case highlights that a rebound in vagal tone and steroid withdrawal may contribute to severe bradycardia during the HAPE recovery phase, warranting heightened clinical vigilance.

## Introduction

HAPE is a well-known acute complication of rapid ascent, characterized by hypoxic pulmonary vasoconstriction and non-cardiogenic edema. While its acute management is standardized, the recovery phase is less well-characterized and is often assumed to be uneventful. We report a unique case of a healthy male who developed severe, symptomatic sinus bradycardia during recovery from successfully treated HAPE. This dramatic shift from initial tachycardia to a heart rate of 37 bpm occurred after discontinuing corticosteroids and diuretics, highlighting the recovery period as a potential window of vulnerability for autonomic dysfunction and serious arrhythmias. This case underscores the need for sustained vigilance beyond the resolution of pulmonary symptoms.

## Case presentation: from HAPE to unexpected bradycardia

**Figure 1:**
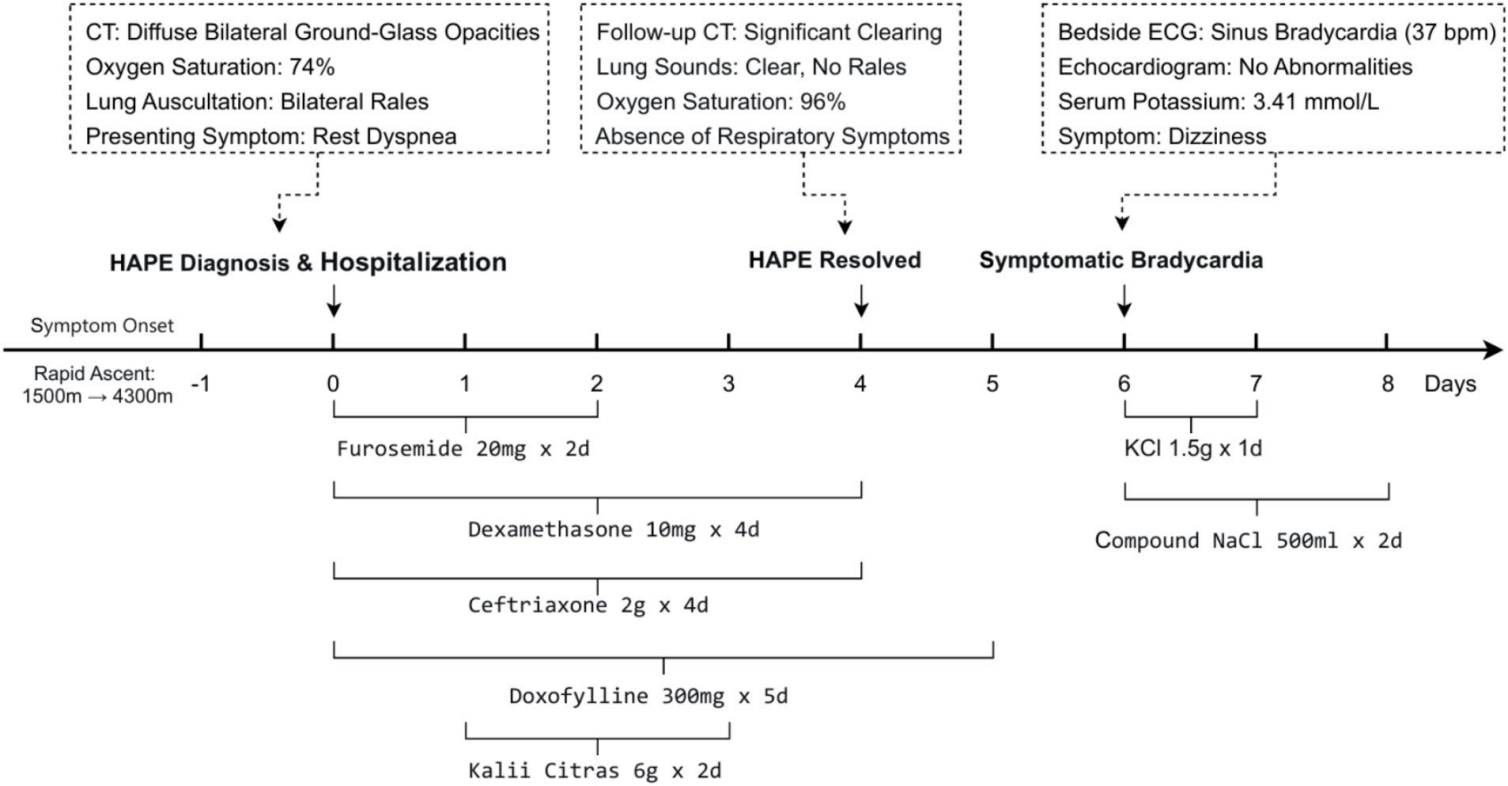
Clinical timeline from HAPE to symptomatic bradycardia and recovery.

A 28-year-old male with no significant past medical history presented to our temporary high-altitude medical station due to severe dyspnea. He had ascended rapidly by air from 1,500 meters to 4,300 meters for work. Shortly after arrival, he reported a worsening cough and shortness of breath, which progressed to dyspnea at rest by the following day (Figure 1).

**Figure 2:**
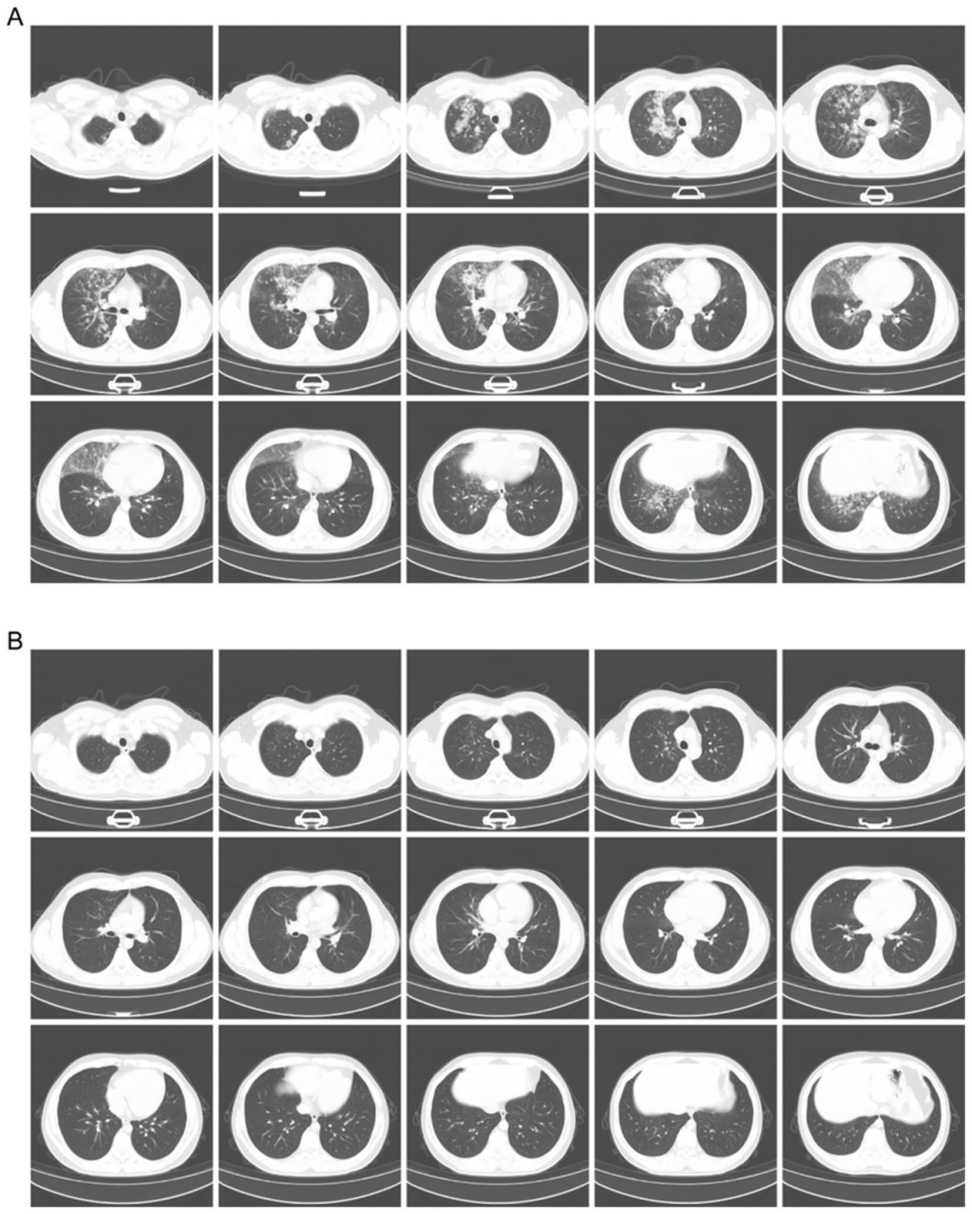
Comparative chest CT imaging before and after treatment for HAPE. **(A)** Admission CT (day 0) reveals extensive bilateral ground-glass opacities with a peri-hilar and peripheral predominance, accompanied by coarse pulmonary markings, reticulations, and areas of traction bronchiectasis, consistent with diffuse interstitial involvement and highly suggestive of HAPE. **(B)** Post-treatment CT (day 4) reveals significant clearing of the infiltrates, illustrating the rapid radiological response to therapy.

**Figure 3:**
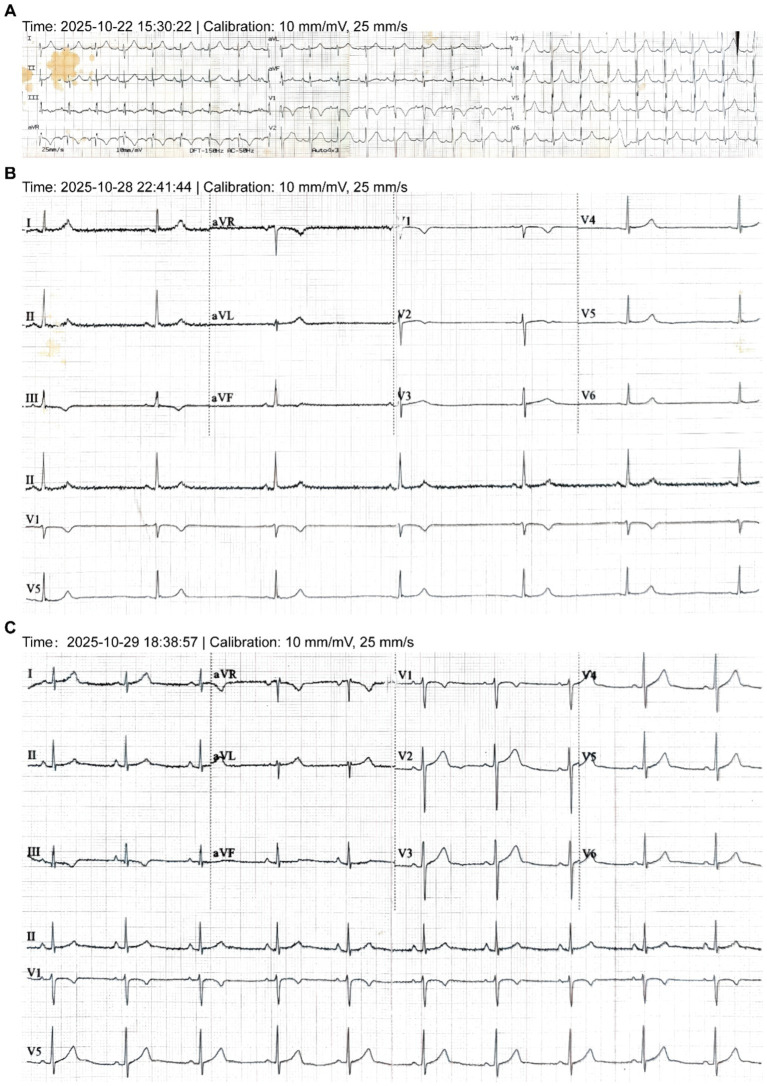
Evolution of electrocardiographic findings. **(A)** Sinus tachycardia (96 bpm) recorded at admission. **(B)** Symptomatic sinus bradycardia (37 bpm) captured on Day 6. **(C)** Restoration of sinus rhythm (59 bpm) following therapeutic intervention. All tracings are shown at the standard calibration of 10 mm/mV and 25 mm/s.

- Initial Presentation and Diagnosis of HAPE: Upon admission (Day 0), the patient was in severe respiratory distress. Vital signs revealed critical hypoxemia with an oxygen saturation of 74%, tachycardia (96 bpm), and a blood pressure of 98/77 mmHg. Physical examination confirmed bilateral pulmonary rales. Laboratory studies were significant for leukocytosis (12.0 × 10^9^/L) and serum potassium (3.81 mmol/L). A chest CT scan revealed bilateral patchy infiltrates, which were characteristic of acute high-altitude pulmonary edema (HAPE, Figure 2A), thereby confirming the diagnosis.
- Successful Treatment and Initial Recovery: The patient was initiated on a standard HAPE treatment protocol, which included intravenous furosemide (20 mg daily for 2 days), dexamethasone (10 mg daily for 4 days), and doxofylline (300 mg daily for 5 days), along with oral potassium citrate (2 g three times daily for 2 days, total 12 g) and prophylactic antibiotics. Following this regimen, his clinical condition improved markedly. A follow-up chest CT on Day 4 demonstrated near-complete resolution of the pulmonary edema (Figure 2B), and discharge was planned.
- Unexpected Clinical Deterioration: Symptomatic Bradycardia: On the second day after the discontinuation of diuretics and corticosteroids (Hospital Day 6), the patient developed a new-onset headache and was found to have profound symptomatic sinus bradycardia with a heart rate of 37 bpm (Figure 3B). This represented a dramatic shift from his initial presentation tachycardia (96 bpm) (Figure 3A). The evolution of these key signs and objective findings across critical time points is summarized in Table 1.

**Table 1:** Evolution of clinical findings from HAPE to bradycardia and recovery.

Clinical parameters	On admission (day 0)	Upon HAPE resolution (day 4)	Onset of symptomatic bradycardia (day 6)	After bradycardia resolution (day 7)
Symptoms	Cough Wheezing Dyspnea	Resolution of respiratory symptoms	Dizziness	Asymptomatic
Physical signs	Bilateral wet rales	No significant rales	HR: 35–40 bpm Regular rhythm	HR: 60–65 bpm Regular rhythm
Oxygen saturation (%)	74%	96%	95%	97%
Heart rate (bpm)	96	70	37	59
Blood pressure (mmHg)	98/77	100/60	117/78	108/62
Serum potassium (mmol/L)	3.81	–	3.41	4.81
Serum magnesium (mmol/L)	1.10	–	1.20	1.11

### Final diagnoses

- Acute Phase: High-altitude pulmonary edema.
- Recovery Phase: Symptomatic sinus bradycardia.

### Assessment and exclusion of secondary causes

Upon the emergence of bradycardia, a focused evaluation was conducted to rule out common secondary causes. A detailed medication and exposure history revealed no use of bradycardia-inducing agents in the preceding month except for ibuprofen prior to admission. Following descent, comprehensive evaluation at a tertiary center was performed. A 24 h Holter monitor documented a minimum heart rate of 31 bpm, a 24 h average heart rate of 55 bpm, and a longest R-R interval of 2.078 s, with no other significant arrhythmias. Thyroid function tests and cardiac biomarkers were within normal limits. These objective findings support the diagnosis of a transient, context-driven autonomic dysfunction.

### Management and outcome of bradycardia

Management shifted abruptly from HAPE to the life-threatening bradycardia. The patient was placed on continuous cardiac monitoring and supplemental oxygen. He received intravenous potassium chloride (1.5 g) via an infusion pump, diluted in 500 mL of normal saline (final concentration ≈ 0.3%), at a controlled rate of approximately 5 mL/min, along with intravenous fluid resuscitation using compound sodium chloride injection (500 mL daily for 2 days). A comprehensive panel of serum electrolytes was obtained approximately 12 h after initiating this intravenous potassium therapy. His heart rate recovered to a stable range of 50–67 bpm within 24–48 h (Figure 3C).

### Follow-up and outcomes

Subsequent follow-up revealed no recurrence of cardiac symptoms. A 24 h Holter monitor performed at a tertiary center showed no significant arrhythmias, confirming the transient nature of the event. Two weeks after discharge, a telephone follow-up confirmed that the patient remained asymptomatic, with self-reported heart rates ranging from 65 to 110 bpm during daily activities, further supporting a full and sustained recovery.

## Discussion

During the acute phase of HAPE, severe hypoxia and stress lead to extreme sympathetic activation, resulting in compensatory tachycardia (1, 2). The profound hypoxia triggers intense pulmonary vasoconstriction, significantly increasing pulmonary vascular resistance and right ventricular afterload. Upon rapid correction of hypoxia and pulmonary edema with effective treatment, the abrupt withdrawal of sympathetic tone, particularly after furosemide cessation and stabilization of volume status, may lead to a relative predominance of vagal tone (3, 4). Concomitantly, the rapid resolution of hypoxia leads to a sudden decrease in pulmonary vascular resistance and right ventricular load. This acute shift in cardiopulmonary hemodynamics may further perturb the autonomic balance, potentially via cardiopulmonary baroreceptor mechanisms, and contribute to the depression of sinoatrial node automaticity. This can profoundly depress the automaticity of the sinoatrial node, causing a sharp decrease in heart rate. In our patient, the heart rate was 96 bpm at admission during the acute HAPE phase. The symptomatic bradycardia occurred on day 6, during the recovery phase after discontinuing primary medications. This temporal pattern strongly supports the hypothesis of autonomic rebound as a primary mechanism.

Corticosteroid-induced bradycardia has been previously documented. For instance, Alvin Oliver Payus et al. (5) reported a case of severe sinus bradycardia in a patient with lupus nephritis following high-dose IV hydrocortisone. Similarly, Mohammed Ahmed et al. (6) described bradycardia in a Crohn’s disease patient after hydrocortisone administration. Our patient received 10 mg of dexamethasone daily for 4 days. Unlike previous reports, bradycardia occurred 2 days after discontinuation. We postulate that short-term, high-dose exogenous glucocorticoids may have transiently suppressed the hypothalamic–pituitary–adrenal (HPA) axis. The subsequent withdrawal led to a state of relative endogenous cortisol insufficiency. Given cortisol’s role in maintaining vascular tone and potentiating catecholaminergic effects, its abrupt decline may have reduced basal sympathetic tone and vascular responsiveness, synergizing with vagal rebound to promote bradycardia. It must be acknowledged, however, that serum cortisol levels were not measured in our patient during the bradycardic episode, which limits the direct biochemical confirmation of this hypothesis.

Hypokalemia significantly affects myocardial excitability. While mild hypokalemia often presents with tachycardia, severe depletion can depress automaticity (7). The mechanism involves altered myocardial membrane potentials, reducing the rate and amplitude of phase 0 depolarization, which can decrease the excitability of sinoatrial pacemaker cells (8). Our patient’s serum potassium dropped from 3.81 mmol/L on admission to 3.41 mmol/L on the day of the bradycardic event, despite oral potassium supplementation. This significant hypokalemia likely exacerbated the bradycardia by further depressing SA node function in the setting of already elevated vagal tone.

This case underscores that the recovery phase from HAPE constitutes a vulnerable window for profound autonomic instability, during which severe bradycardia can occur. This paradoxical shift from near-universal acute-phase tachycardia to profound bradycardia upon recovery suggests a critical and under-recognized transition period. The pathophysiology likely involves a triad of extrinsic, reversible factors acting in concert: abrupt sympathetic withdrawal following rapid hypoxia correction, relative endogenous cortisol insufficiency after exogenous steroid cessation, and hypokalemia. The concurrent precipitous drop in pulmonary vascular resistance after HAPE resolution may further perturb cardiopulmonary coupling and baroreceptor feedback, favoring vagal predominance. Although no identical cases are reported, this multifactorial mechanism aligns with reversible sinoatrial node dysfunction seen in other critical illnesses. Consequently, in resource-limited high-altitude settings, clinical vigilance must extend well beyond the resolution of pulmonary symptoms. Close monitoring of heart rate, blood pressure, and electrolytes remains crucial even after primary medications are discontinued and imaging shows significant improvement.

## Patient perspective

The patient reported having minimal prior understanding of high-altitude pathology. He described the sequential onset of severe dyspnea followed by acute dizziness during recovery as a profoundly alarming experience. He expressed sincere appreciation for the coordinated care provided under resource-limited conditions. This episode has fundamentally altered his risk perception regarding high-altitude travel.

## Data Availability

The original contributions presented in the study are included in the article/supplementary material, further inquiries can be directed to the corresponding authors.
